# Research Development in Silica Aerogel Incorporated Cementitious Composites—A Review

**DOI:** 10.3390/polym14071456

**Published:** 2022-04-02

**Authors:** Agnieszka Ślosarczyk, Andrii Vashchuk, Łukasz Klapiszewski

**Affiliations:** 1 Institute of Building Engineering, Faculty of Civil and Transport Engineering, Poznan University of Technology, Piotrowo 3, PL-60965 Poznan, Poland; andrii.vashchuk@doctorate.put.poznan.pl; 2 Institute of Chemical Technology and Engineering, Faculty of Chemical Technology, Poznan University of Technology, Berdychowo 4, PL-60965 Poznan, Poland

**Keywords:** silica aerogel, insulating materials, lightweight cementitious composites, mechanical and insulating properties, interfacial transition zone, porosity

## Abstract

This paper presents an analysis of research results for silica aerogel cement composites over the past twenty years. Recently, two trends in the development of these composites have been noted, towards structural applications and towards ultralight composites for coatings and renders. Ongoing research shows that important aspects of cementitious composites with good mechanical performance are the proper selection of aggregates and improved adhesion at the silica aerogel–cement binder interface, which will guarantee high compressive strength with the lowest possible thermal conductivity. The best physicomechanical performance of aerogel cement composites with low thermal conductivity below 0.03 W/(m·K) was obtained when cenospheres and aerogel were used in a weight percentage of 5%. In turn, the prerequisites for using aerogel cement composites as coatings for energy-efficient building façades are the use of large amounts of silica aerogel as a substitute for lightweight aggregates or the selection of an optimal composition of lightweight aggregates and aerogel, ensuring the lowest possible thermal conductivity coefficient. Other important standpoints are water transport and moisture protection of the silica aerogel-based coatings. Therefore, in recent years, more and more elements of the hygrothermal performance, porosity and durability of silica aerogel cement composites have been developed. The article also points out the weaknesses of the application of silica aerogel in the cement matrix, the most important of which are the lack of adhesion at the boundary of the aerogel–cement binder, the increased porosity of the composite, the high water absorption capacity and the significant decrease in compressive strength with large volumes of silica aerogel. Solving these issues will certainly contribute to the wider applicability of these materials in the construction industry.

## 1. Introduction

Approximately 40% of all energy consumption and 36% of all CO_2_ emissions in Europe stem from residential and industrial buildings due to inefficient insulation materials and systems. To address this, the Energy Performance of Buildings Directive (EPBD) 2010 required that, by the year 2020, almost all new constructions were to be “Nearly Zero Energy Buildings”. Unfortunately, this criterion has not been met in many European countries and the time to achieve restrictive requirements has been extended for the coming years. In addition, there is still a problem with existing buildings and how to upgrade them under current insulation conditions. This performance may only be achieved either by installing extremely thick conventional insulation materials, by sacrificing living spaces or by using materials of very low thermal conductivity and density without limitations in compressive strength. For this reason, novel materials combining low thermal conductivity and density with high compressive strength should be developed for building applications [1,2].

Some of the most important challenges in today’s construction industry are ensuring the safety, durability and reliability of buildings. An essential condition in overcoming these complex issues is developing new building materials that have high strength and durability and, at the same time, are safe for human health and for the natural environment. The application of nanotechnologies in construction enables the design and modification of material structures so that we receive a product with the expected strength and some special directional properties, such as resistance to external physical and chemical factors, as well as being self-cleaning, antibacterial or self-repairing [3,4,5,6,7,8,9,10,11]. The EU lists this area of research in the document called “Roadmap 2014” as a key one. Indeed, according to research center predictions, nanotechnology will play increasingly larger roles within the next several dozen years in the production of innovative materials for construction [12,13,14]. Nanomaterials that may find wider use in the construction industry are silica aerogels and their composites, including those that are cementitious-based [15,16,17,18].

The aim of the article is to present the most important trends in the synthesis and physical properties of cement composites modified with silica aerogel granules. The article covers three topics: firstly, the properties and applications of silica aerogel; then, the main achievements of recent years in aerogel cement composites; and lastly, the most important aspects related to the durability and performance of these composites. The article shows that the recent years of development of aerogels in cement composites, especially based on lightweight aggregates, can make an important contribution to reducing thermal conductivity and increasing the applicability of silica aerogel in the construction industry. However, scientists still face problems associated with this material, such as low adhesion to cement binders or reduced strength parameters, and point out the need to test the durability and performance of silica aerogel composites over a longer service life.

## 2. Silica Aerogel—Synthesis and Properties

Silica aerogels are made of 95% air, the rest being silica crystal (SiO_2_). They have an open porous structure that is composed of particles with diameter less than 10 nm and pores smaller than 50 nm. These properties give silica aerogels a very low density of 0.03–0.35 g/cm^3^, with fairly high BET surface areas (500–1200 m^2^/g). They also have a thermal conductivity coefficient below 0.02 W/(m∙K), a low dielectric constant of 1.1, speed of sound of 100 m/s and refractive index within the range 1.00–1.08 [19,20,21]. The exceptional properties of silica aerogels allow for their use in numerous industries; they can be employed as thermal and acoustic insulators [22,23,24,25,26,27,28], catalytic converters, carriers of active substances [19,29,30], absorbents of gases, liquids or energy and sensors [31,32,33,34,35].

Silica aerogel was invented in the 1930s, by Stephan Kistler, but only the last twenty years have brought considerable interest in this material [19]. The intensive development of research on silica aerogel has been initiated by a group that has used organic silica compounds such as TMOS (tetramethylorthosilicate) or TEOS (tetraethylorthosilicate) as precursors. Silica aerogels are synthesized in three stages by the sol–gel process. In the first stage, a gel is prepared by a precursor solution (silica source) with the addition of a catalyst. In the second stage, the gel is aged either in water or the mother solution. The aim of aging is to consolidate the gel and minimize the shrinkage of the gel during drying. The drying step can be realized mainly through three ways, namely supercritical at high or low temperatures, and ambient pressure drying. In the high-temperature supercritical drying method, the gel is put together in an autoclave with an alcohol such as methanol or ethanol, and the temperature is slowly increased until the supercritical temperature and pressure are reached. The fluid is then removed at constant temperature. In the low-temperature supercritical drying method, the alcohol present in the pores of the gel is replaced with another liquid, such as liquid CO_2_, which has a critical point close to ambient temperature. Here, the wet gel is placed in an autoclave, and liquid CO_2_ is pumped in at 4–10 °C until the pressure reaches 100 bar. Subsequently, the solvent inside the pores of the gel is extracted, and the autoclave is heated close to 40 °C to reach the supercritical conditions of CO_2_ [36,37].

Even though the supercritical drying process is the most common process, and is the most suitable for monolithic aerogel production, the cost and safety risks, especially for high-temperature supercritical drying, are limitations. In the ambient pressure drying process, the water–alcohol mixture in the pores of the gel is first exchanged for a water-free solvent. The surface modification is then reacted with a silylating agent so that the Si–OH groups are replaced by methyl silyl groups. The substitution of the H from the Si–OH groups by the hydrolytically stable Si–R groups hinders the adsorption of water, and the aerogel becomes hydrophobic. After solvent exchange, evaporative drying takes place [20,38,39]. The ambient pressure drying procedure is advantageous when compared to the supercritical drying in terms of cost and safety since it does not require high pressures or expensive high-pressure equipment. Nevertheless, there are additional chemicals and solvents employed. Therefore, to make this process suitable for commercialization, minimum amounts of solvent should be used with a minimum number of solvent exchange steps. Nevertheless, laboratory-synthesized silica aerogels are very fragile for sole application in the building sector. Thus, further research should focus on improving the silica aerogel’s strength and incorporating it into stronger organic or inorganic, etc., matrixes [40,41,42].

By means of the proper selection of particular parameters of synthesis, precursor and modification method, it is possible to alter the final structural and mechanical properties of silica aerogels at an early stage of the synthesis.

The strength and stiffness of the gel can be improved at the stage of ageing the gel by dissolving and repeatedly precipitating silica from the surface of particles onto the borderline particle–particle and connecting and/or precipitating oligomers that were unreacted during gelling. Another method assumes adding extra amounts of precursor and co-precursor to the solution before and after the moment of gelation, so that it builds into the structure of the gel and, thus, reinforces it [43,44,45].

Apart from altering the parameters of the synthesis, the mechanical properties of silica aerogels can be modified by incorporating various additives into their structure, e.g., nanoparticles and metal nano oxides, or by applying reinforcement in the form of short structural fibers or fiber mats [46,47,48,49,50,51,52]. There is also research carried out on covering the surface of silica aerogels with polymers [53,54,55,56]. This action is taken before the stage of drying the gel; as a result, the surface of the silica aerogel is covered with a layer of polymer that increases the resistance of silica structure to breaking. In addition to the above-mentioned strengthening of the aerogel structure with fibers, an alternative solution may also be to introduce silica aerogel into more durable and stronger structures with a low thermal conductivity coefficient, such as a polymer matrix or concrete.

Concrete, in comparison with other building materials such as stone or steel, is characterized by a relatively low thermal conductivity coefficient, reaching maximum values of roughly 2.0 and 2.5 W/(m·K) for average concrete with a density from 2200 to 2400 kg/m^3^ and for reinforced concrete (with steel bars), respectively [57]. The thermal conductivity coefficient of concrete can be easily lowered via air entrainment or the application of a lightweight aggregate characterized by high porosity and a low thermal conductivity coefficient. Unfortunately, very often, high porosity in concrete and lightweight aggregates leads to a significant decrease in composite compressive strength—down to a few Mpa—and eliminates such solutions in terms of construction potential. In addition, the application of a modification of the cement binder with polymers may improve the adhesion of the binder to the aggregate and thus enhance the mechanical parameters of the composite [58,59]. There is, however, a group of lightweight aggregates that enable higher strength parameters to be obtained with a relatively low thermal conductivity coefficient [60,61,62,63,64,65,66,67] (see Table 1).

Among these aggregates, microspheres have the best strength and insulation parameters. Microspheres (cenospheres) are hollow silica and alumina spheres with a diameter of less than 500 µm that are produced as a by-product of coal combustion in thermal power plants. The most important characteristics of microspheres are low bulk density (about 400 kg/m^3^), low thermal conductivity 0.1 W/(m·K) at room temperature, low coefficient of thermal expansion (6.13 × 10^−6^ 1/K) and high melting temperature above 1200 °C (which gives them high temperature resistance) [65,66]. Studies have shown that the use of cenospheres with diameters ranging from 300 to 600 µm in cement composites leads to very high strength parameters (with compressive strengths reaching approximately 40–70 MPa), while low densities are maintained and thermal conductivity coefficients range from 0.29 to 0.60 W/(m·K) [62,64,65,66].

## 3. Composition, Mechanical and Insulating Properties of Cementitious Composites with Silica Aerogel Granulate

### 3.1. Overview of Cementitious Composites with Silica Aerogel

It can be noticed that, during the last decade, studies on cementitious composites with silica aerogel were performed with two main concepts in mind: the incorporation of silica aerogel into structural concretes or mortars, or as lightweight composites that could be used as structural and insulation materials (see Table 2).

It should be mentioned that some of the investigators use a combination of the words ‘concrete’ and ‘lightweight composite’ for the description of their cementitious composites; therefore, it seems to be reasonable for order purposes to introduce a catalogue conception in the future that could be used globally, thus avoiding uncertainty in material requests.

The conducted literature review reveals that researchers in the field have chosen various approaches and applied various techniques that should be seen as positive factors in the global development of building engineering and science.

Gao et al. [67] investigated the influence of aerogel content on the physical and mechanical properties of concrete. For the purpose of the study, concrete samples were prepared with an aerogel content of 0%, 20%, 40% and 60%. Results showed that samples with 60% of aerogel had the best mechanical and thermal conductivity properties for the experiment. Gao et al. recorded that samples with 60% of aerogel had thermal conductivity of 0.26 W/(m·K) and compression strength of 8.3 MPa. Moreover, a follow-up investigation of stability of the aerogel particles in the concrete samples at the microscopic scale showed that the aerogel particles were mixed fairly well with the cement before and after adding water. Moreover, microscope images showed uniform dispersion of aerogel particles within the cement matrix [67].

Fickler et al. [69] conducted an experimental study on the impact of heat treatment on the mechanical properties of high-performance concrete with silica aerogel amendment. For the purpose of the study, a high-performance concrete recipe was used and samples were exposed to different heat conditions (different temperatures and time sessions). Results indicated negligible effects of the heat treatment of samples on their mechanical strength and thermal conductivity. Compression and flexural strength for the samples with 60% of silica aerogel were 8.3 MPa and 1.2 MPa, correspondingly, whereas the recorded thermal conductivity of the samples was 0.19 W/(m·K) [69].

Ng et al. [74] investigated the utilization of calcined smectite-enriched clay as a partial binder in the aerogel-incorporated mortar. Results indicated a reduction in thermal conductivity of up to 20% with maintained mechanical strength in samples with 60% of silica aerogel [74]. In another study, Ng et al. [75] investigated the impact of various storage and curing conditions on the mechanical strength and thermal conductivity of ultra-high aerogel concrete samples. The samples were exposed to different temperature and moisture conditions during different periods of time. The results indicated a negligible increase in the compression and flexural strength of the samples. Deviation in the results was recorded, however, as, in some samples, the effect was the opposite. For samples with 60% of aerogel, compression and flexural strength were 14.5 MPa and 3.75 MPa, correspondingly, and thermal conductivity was 0.3 W/(m·K) [75].

Hanif et al. [79] analyzed the mechanical and thermal insulating properties of lightweight composites with silica aerogel and fly ash cenospheres. In these, relatively low amounts of silica aerogel (1–5%) were used for sample preparation. Samples with 5% of silica aerogel demonstrated a decrease in mechanical strength, whereas thermal conductivity was reduced from 0.41 W/(m·K) (ref. sample) to 0.26 W/(m·K). A mercury porosimetry test indicated the agglomeration of aerogel particles in samples with 4% and 5% of silica aerogel [79].

### 3.2. Density and Mechanical Properties

**Figure 1:**
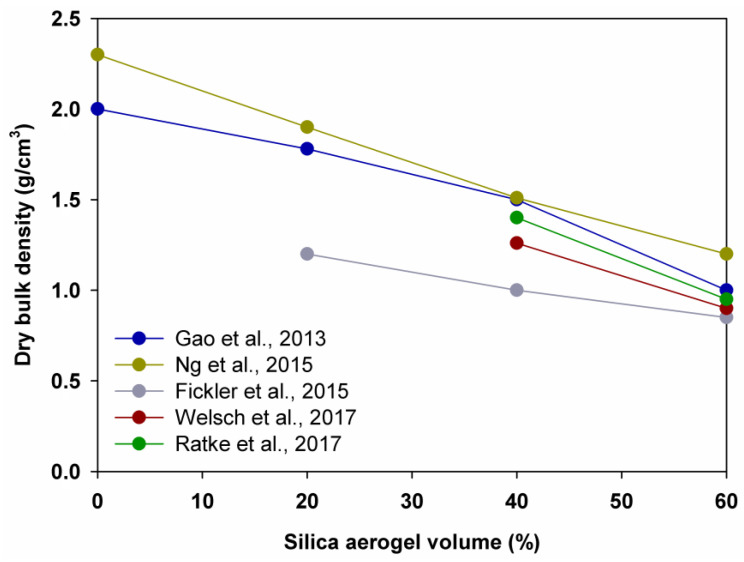
Density of cementitious composites with silica aerogel. Adapted from [67,68,69,70,88].

According to the literature, the density of the cementitious composites with silica aerogel can vary from 1.78 to 0.70 g/cm^3^, whereas conventional concretes and mortar have density from 2.40 to 2.70 g/cm^3^. Studies show that the density parameter of cementitious composites can be influenced by the silica aerogel amount, type of recipe or mixing techniques (see Figure 1).

**Figure 2:**
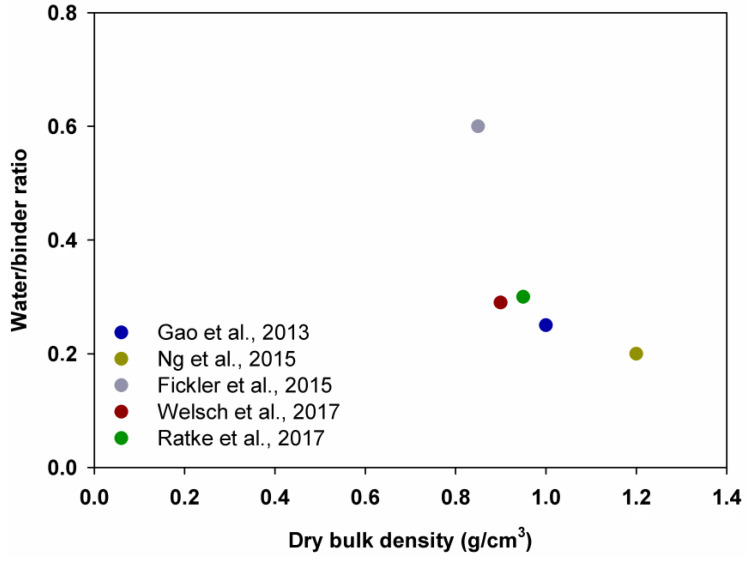
Relationship between water/binder ratio and dry bulk density (samples with 60% of aerogel). Adapted from [67,68,69,70,88].

Gao et al. [67] recorded that the density decreased by 11.0%, 15.7% and 33.0% as the volume of the aerogel increased from 0% to 20%, 20% to 40% and 40% to 60%, whereas Ng et al. [68] observed that the density decreased by 17.4%, 21.6% and 20.5%, correspondingly. Furthermore, Ratke et al. [88] and Welsch et al. [70] prepared samples with 40% and 60% of silica aerogel and noted that the density decreased by 28.6% and 32.0%, correspondingly, while Fickler et al. [69] and Gao et al. [67] claimed a reduced density of concrete samples by 16.6% and 33.0% when the amount of silica aerogel was increased from 40% to 60%. A positive correlation between density and water/binder ratio was inferred by all authors (see Figure 2).

**Figure 3:**
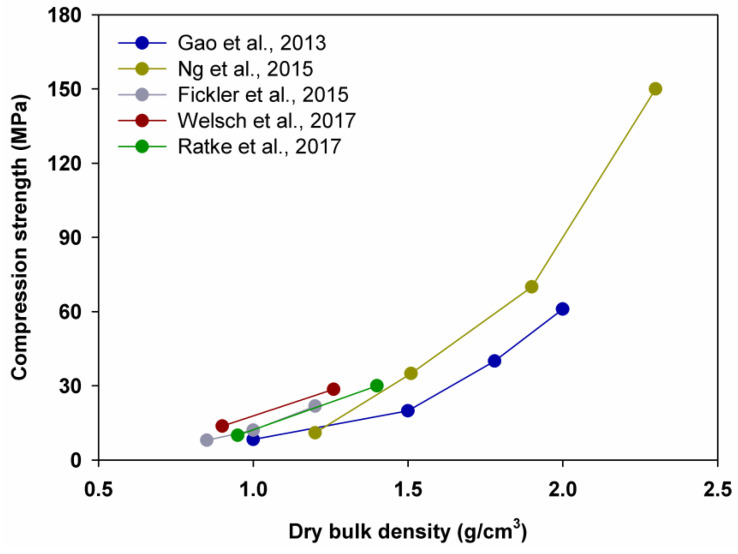
Relationship between dry bulk density and compression strength. Adapted from [67,68,69,70,88].

The presented correlation shows that a higher water/binder ratio of the samples corresponds with a smaller dry bulk density. Moreover, a lower density of composites leads to a decrease in compressive strength, as shown in Figure 3. A decrease in the density and mechanical strength of the samples due to increased porosity because of air voids around silica aerogel granules was confirmed by microscope scans and mercury porosimetry tests [67,68].

**Figure 4:**
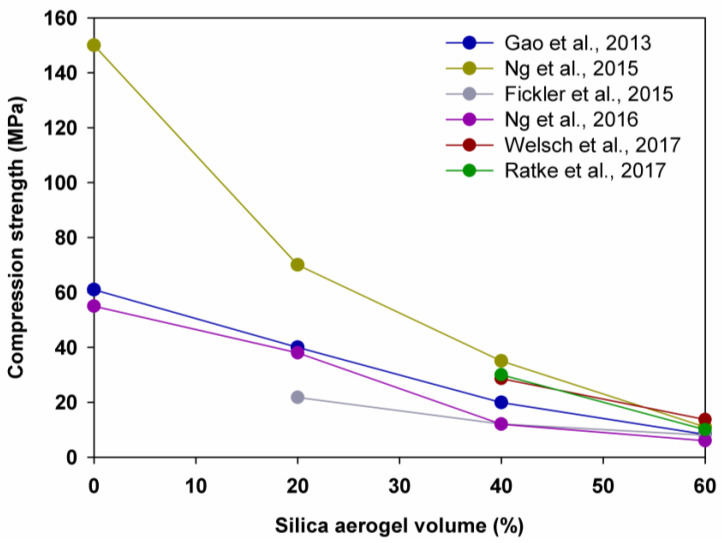
Relationship between compression strength and silica aerogel volume. Adapted from [67,68,69,70,88].

Gao et al. [67] demonstrated a reduction in compression strength by 34.4%, as the aerogel content was increased from 0% to 20%; by 50.3%, as the aerogel content was increased from 20% to 40%; and by 58.3%, as the aerogel content was increased from 40% to 60%. Ng et al. [68], in turn, observed a reduction in the compression strength by 53.3%, 50.0% and 68.6%, respectively. In addition, Fickler et al. [69], Welsch et al. [70] and Ratke et al. [88] saw reductions in the compression strength by 33.8%, 52.0% and 66.7%, respectively, as the silica aerogel amount was increased from 40% to 60% (see Figure 3 and Figure 4).

**Figure 5:**
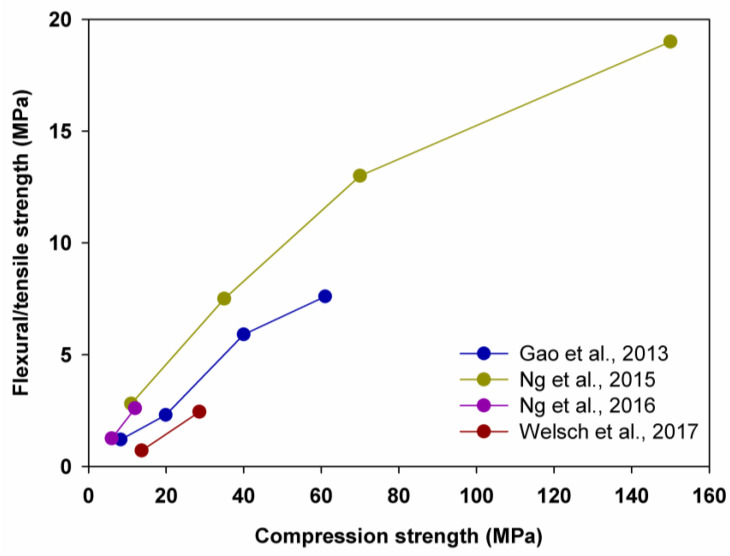
Relationship between compression strength and flexural/tensile strength. Adapted from [67,70,74,75].

Correspondingly, a significant reduction in the flexural/tensile strength was observed (see Figure 5). Gao et al. [67] recorded that the flexural/tensile strength was decreased by 22.4%, 61.0% and 48%, as the amount of aerogel was increased from 0% to 20%, 20% to 40% and 40% to 60%, respectively. Ng et al. [74,75], in turn, indicated that the flexural/tensile strength was reduced by 32.0%, 42.3% and 63.0%, accordingly, while Welsch et al. [70] saw that the flexural/tensile strength was reduced by 71%, as the amount of aerogel was increased from 40% to 60%.

**Figure 6:**
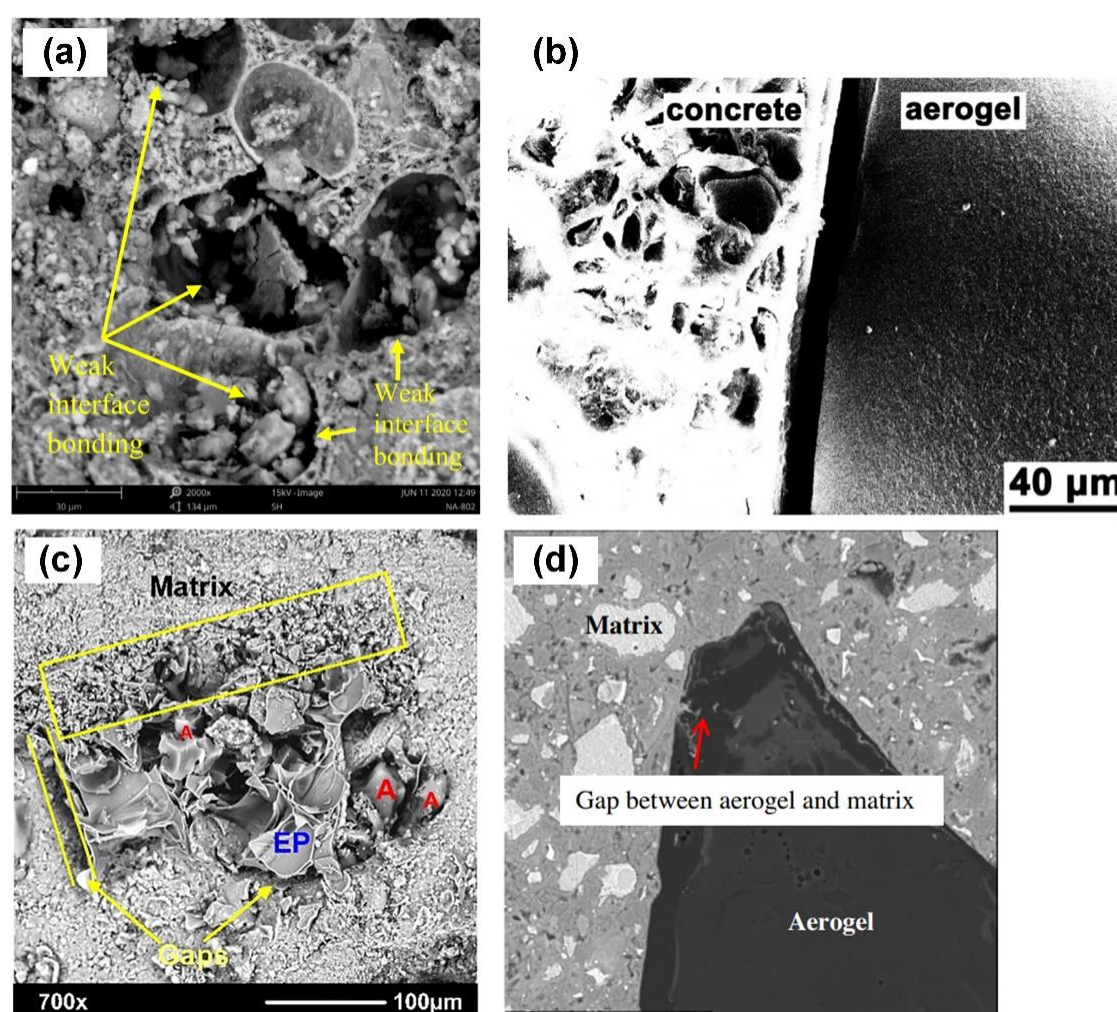
SEM images for cementitious composites with silica aerogel with visible gap between cement paste and silica aerogel. Reproduced with permission [67,68,89,90], respectively, for (**a**–**c**), and (**d**). Copyright 2014, 2015, 2021, Elsevier.

According to the literature data presented, the introduction of silica aerogel in the form of granulate into the cement matrix instead of a traditional aggregate is associated with a simultaneous drastic decrease in compressive strength. Some researchers indicate that the reason for this is the lack of adhesion between the aerogel and cement paste [67,68,76,89,90]. Figure 6 shows the transition zone between aerogel and cement binder and reveals the aforementioned air gap between the two materials. The apparent gap between the aerogel and the cement binder is due to the hydrophobic character of the aerogel surface and is one of the reasons for the deterioration in the mechanical parameters of the composite. Moreover, the brittleness of the silica aerogel itself and increased porosity of the cement matrix also determine the mechanical parameters of the composite. Therefore, future research should focus on improving the adhesion at the silica aerogel–cement binder interface and enhancing the flexibility of the material itself.

One way to improve the adhesion between the silica aerogel and the cement matrix may be to use short fibers in dispersed form. Westgate et al. [91], for example, introduced short polypropylene fibers of 12 and 18 mm length and 20 µm diameter at 0.5 vol.% as a reinforcement for lime plaster with silica aerogel. In this way, they obtained improved fracture toughness and homogeneity of the composite, as the fibers bridged the resulting microcracks and provided integrity between the lime and aerogel [91]. Jang et al., in turn, investigated cementitious composites with carbon nanotubes and silica aerogel [84]. During this experiment, the thermal conductivity of samples with silica aerogel amounts from 0.25% to 2% was measured. The researchers indicated that the thermal conductivity of samples was enhanced, while the mechanical strength changed negligibly due to the presence of the carbon nanotubes and the low amount of silica aerogel amendment. Jang et al. showed that relatively low amounts of silica aerogel in the composite and second aggregate in the form of high-tech or eco material might enhance the thermal conductivity [84]. The compatibility of such a technique was experimentally proven by Adhikary et al. [73]. For their investigation, two groups of samples were prepared. The first was with silica aerogel and expanded glass, and the second incorporated silica aerogel and prefabricated plastic bubbles. The researchers observed increased flowability and better workability of the mixture of both samples in comparison to the standard. In both cases, the compression and flexural strength of the samples increased from 3 MPa to 4 MPa and from 1 MPa to 1.29 MPa, respectively [81].

In turn, Shah et al. clearly indicate that an important issue in the future will be to increase the wettability of the silica aerogel so that good bonding with the cementitious binder can be achieved. This should, accordingly, lead to good mechanical performance and reduced absorbability of the composite [89]. The work of Al Zaidi et al. exemplifies such a solution [72]. In their study on structural concrete, the authors worked on the improvement of the interface between the silica aerogel and cement matrix. A pre-treatment technique of silica aerogel particles with methanol was applied therein. The results demonstrated that pre-treatment led to better blending of the silica aerogel with the cement paste and played an important role in reducing the porosity of the samples. This outcome was confirmed via microscope imagery. Final measurements showed that samples with 60% of silica aerogel had compression strength from 27 MPa to 30 MPa, flexural strength of 3.82 MPa and thermal conductivity ranging from 0.86 W/(m·K) to 1 W/(m·K) [72]. In another experiment, Rostami et al. [85] applied a special treatment to the aggregate. In preparing the samples, the investigators used 5% to 10% of silica aerogel and paraffin-coated recycled aggregate. The results indicated that the thermal conductivity of the samples with 5% and 10% of silica aerogel was reduced by 31–38%, in comparison to the samples without silica aerogel. According to the publication, a reduction in the mechanical strength was not recorded; therefore, additional studies have to be performed in order to prove the applicability of this method in the future [85].

Improved adhesion at the silica aerogel–cement binder interface can also be achieved by modifying the cement binder with chemical admixtures with rheological and adhesion-enhancing properties or by improving the porosity of the microstructure [92,93]. Pedroso et al., for example, modified the cement matrix with a superplasticizer and resin and obtained a homogeneous distribution of aerogel in the cement binder, yielding a state-of-the-art coating material with a very low thermal conductivity of 0.029 W/(m·K) [92]. In contrast, Yoon et al. produced a new foam concrete into which they introduced uniformly distributed silica aerogel [93]. They used two precursors, pure MTMS and a mixture of MTMS and TEOS, to synthesize the silica aerogel. The authors demonstrated that the resulting aerogel filled the pores formed during the foaming of the concrete, resulting in a lightweight concrete structure with a 75% reduction in water absorption and a 30–50% lower thermal conductivity coefficient compared to conventional foam concrete [93].

Recent publications suggest also that the enhancement of the mechanical parameters with retained insulating properties might be achieved by adding other lightweight aggregates along with the aerogel [76,87,90,94,95,96,97,98]. Aggregates used in these solutions include expanded cork, expanded polystyrene, expanded glass or expanded perlite and vermiculite. For example, Morgado et al. used regranulated expanded cork, silica aerogel and expanded polystyrene as aggregates for energy-efficient building façades [87]. They indicated that, in doing so, the weight of the samples was decreased, whereas the compression strength was slightly increased. Jia and Li also proposed an interesting solution by creating a composite aggregate based on perlite soaked in silica aerogel to fill the pores of the lightweight aggregate [90]. Additionally, the adhesion at the aggregate–cement paste interface was enhanced by the presence of microsilica and silanes. Thanks to this solution, good mechanical parameters of the cement composite were achieved, ranging from 3.79 to 14.47 MPa, and low densities ranging from 524 to 951 kg/m^3^. They determined that the thermal conductivity coefficient was approximately 10–30% lower than that of cement composites made of pure expanded perlite [90].

In recent years, in addition to the development of aerogel cement composites for structural applications, lightweight coating materials for façade systems, mainly in the form of renders, have received considerable attention. In these solutions, natural or artificial aggregates are completely replaced by silica aerogel, or the aerogel represents a significant volume share of the aggregates used [99,100,101,102,103]. This results in very low strength parameters, and these mortars reach compressive strengths of several MPa. However, these materials are characterized by low density, so the heat conductivity coefficient for these material solutions reaches values even below 0.2 W/(m·K). An interesting solution is presented in the work of de Fátima Júlio et al. and documents a new approach to the synthesis of cement-based thermal renders, where the natural aggregate (sand) was replaced by silica aerogel with different properties [100]. The authors of this paper independently synthesized silica aerogel varying in degree of hydrophobicity (with and without hexamethyldisilizane modification), using much cheaper and ecologically desirable atmospheric drying, and compared it with a commercial aerogel. In addition, they used an anionic surfactant to improve adhesion at the silica aerogel–cement binder interface. They thus obtained a very low thermal conductivity coefficient of 0.085 W/(m·K) and a low material density of 410 kg/m^3^, for HMDZ-modidied aerogel-based renders in which silica aerogel completely replaced the natural aggregate. These studies show that the future of aerogel cement composites and their wider application will also significantly depend on improvements in the properties of the silica aerogel itself, its flexibility and in its surface functionalization, providing good adhesion to the cement matrix [100].

### 3.3. Insulating Properties of Cementitious Composites with Silica Aerogel

Experimental studies have revealed that silica aerogel granules mixed fairly well with cement. Accordingly, substitution of conventional aggregates with 40% and 60% of silica aerogel enabled a decrease in the thermal conductivity of the samples from 1.90 W/(m·K) to 0.80–0.19 W/(m·K) (see Table 3).

Gao et al. [67], in turn, reported that the thermal conductivity was decreased by 23.7%, 44.8% and 68.75%, as the volume of silica aerogel was increased from 0% to 20%, from 20% to 40% and from 40% to 60%, respectively. Moreover, Ng et al. [68] recorded that the thermal conductivity was reduced by 34.7%, 46.7% and 37.5%, correspondingly, while Ng et al. [74], Welsch et al. [70] and Ratke et al. [88] revealed that increasing the amount of aerogel from 40% to 60% reduced the thermal conductivity by 37.5%, 25.0% and 30.8%, respectively.

Beyond the aforementioned, in various publications, thermal conductivity of 0.2 W/(m·K) was achieved when the amount of silica aerogel was 60% and higher (see Table 3). Of note, several authors suggest that it might be useful to evaluate the criterion separately for each group of cementitious composites.

**Figure 7:**
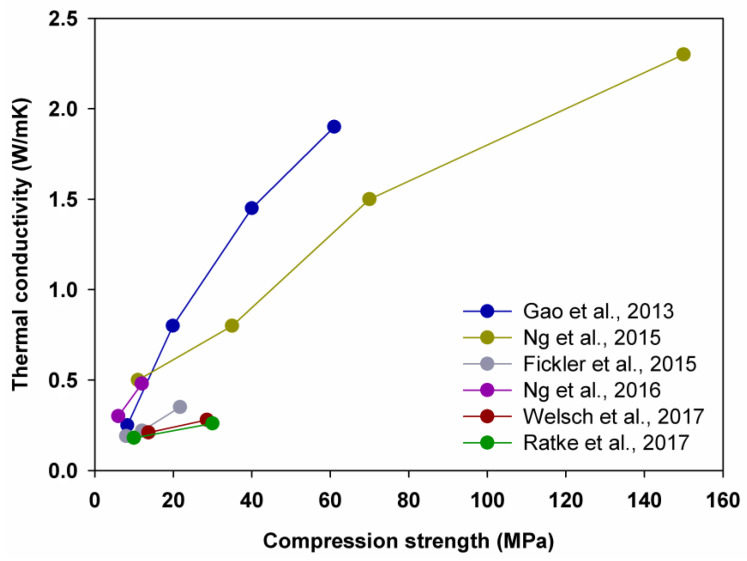
Relationship between thermal conductivity and compression strength. Adapted from [67,68,70,74,75,88].

The analysis of the results so far clearly shows the dependence of the thermal conductivity coefficient value on the material density. Lower material density results in a decrease in the thermal conductivity coefficient, as well as a drastic decrease in the compressive strength, as shown in Figure 7. This is due to the increased porosity of the cement composites, which significantly depends on the amount of silica aerogel introduced into the structure. The effect of the porosity of aerogel cement composites on their physicomechanical parameters, mainly their compressive strength and thermal conductivity coefficient, has been studied by many researchers [68,89,104,105,106]. According to Shah et al., silica aerogel, in introducing a high degree of hydrophobicity into the structure of the cement matrix, contributes to an increase in the number of macropores, which in turn causes a decrease in the density of the material by 35% and compressive strength by 76% while enhancing the absorbability and water absorption rate of the composite [89]. Changes in the porosity of lightweight cement composites as a function of the addition of silica aerogel and air-entraining admixture and aluminum powder was also investigated by Strzałkowski and Garbalińska [105]. Their work demonstrated that the highest porosity of the material and thus the lowest thermal conductivity for cement composites was achievable with silica aerogel and air-entraining admixture. In all cases, a significant decrease in compressive strength was observed [105]. Nevertheless, the study of Ng et al. shows that by proper choice of the individual components of the cement composite, selection of the ratio of silica aerogel to the remaining aggregate and by proper curing of the specimens, both the strength parameters and thermal conductivity coefficient can be significantly affected [74,107].

In such work, a strength of 19 MPa and a thermal conductivity of 0.4 W/(m·K) was obtained at 60 vol.% of silica aerogel, while lower thermal conductivity coefficients and compressive strength were gained at higher aerogel volumes above 70 vol.%. In this case, thermal conductivity between 0.1 and 0.2 W/(m·K) was achieved and the compressive strength was approximately 5 MPa.

It has been shown that the mechanical and insulating parameters depend to a large extent on the temperature and humidity of the storage process and the curing of the specimens. Herein, an increased storage and curing temperature brings about increased hydration of the cement binder, which yields an increase in the strength parameters and a decrease in the thermal conductivity coefficient. Bostanci et al. [107] investigated the influence of the curing of cement composites with silica aerogel on porosity, as well as the thermal and mechanical parameters. In this work, aerogel was added between 0.3 and 1 wt.%. The samples were then matured under different conditions, under water, wetting–drying and MgSO_4_ curing, The outcome was that significantly higher compressive and flexural strengths were obtained with wetting–drying and MgSO_4_ maturation, with significantly better performance at higher temperatures. In this way, satisfactory structural parameters can be formed for precast elements while maintaining higher porosity and lower thermal conductivity, as compared to conventional concrete elements [107].

## 4. Durability and Performance of Silica Aerogel-Based Cementitious Composites

Increased porosity of the cement matrix due to the presence of silica aerogel and poor adhesion at the silica aerogel–cement paste interface are key factors affecting the durability of cement composites. Therefore, it seems very important to study the durability of these materials and the performance over a long service life. There have been a few publications in recent years in which the authors extended the scope of their study and evaluated the durability, fire resistance and exposure to solar radiation of silica aerogel-based cementitious composites [108]. Nevertheless, all authors agree that this is a direction for future research on these materials.

Stefanidou and Pachta [76], for example, looked into the fire resistance properties of cement-based mortar with silica aerogel and perlite. For the purpose of the study, 20% of the aggregate was replaced with silica aerogel and perlite. After curing, the samples were exposed to elevated temperatures of 800–1000 °C. The investigators indicated that the samples containing both silica aerogel and perlite maintained mechanical strength before and after exposure to high temperatures, whereas samples without silica aerogel did not maintain residual mechanical strength [76].

From the results of the above-mentioned work, it can be inferred that the fire-resisting properties of silica aerogel might find application in the field of insulation materials. Such properties were investigated by several researchers [75,86,87,108,109]. In the publication of Ismail et al. [86], for example, the investigators presented an experimental study on the energy efficiency of cement-based thermal cladding with silica aerogel amendment. For the purpose of the research, mechanical strength, thermal conductivity and exposure to solar radiation were analyzed, and the insulating capability of the renders was tested under exposure to various climate conditions. The researchers indicated that suitable energy efficiency and insulating capability were achieved [86]. Morgado et al. [87], in turn, investigated the durability parameters of thermal renders with silica aerogel and other eco-friendly materials. The scope of the experiment was to expose samples to long-duration freeze/thaw cycles and to hygrothermal accelerated aging cycles. Between and after exposures, mechanical strengths and thermal conductivity were measured. According to the final results, the long-duration freeze/thaw cycle and the accelerated aging cycle led to an increase in the compression strength of renders with re-granulated cork and renders with expanded polystyrene. Moreover, renders with silica aerogel maintained their mechanical strength, whereas the thermal conductivity of the renders with silica aerogel was reduced from 0.20 W/(m·K) to 0.09 W/(m·K) before and after exposure to several freeze/thaw cycles [87]. The work of Morgado et al. shows the significant relationship between the porosity of the cement matrix and the thermal–moisture properties of potential coating materials.

Hygrothermal testing under different climatic conditions has also been studied by other researchers [110,111,112]. The results of Sakiyama et al., for instance, showed high water absorption in the analyzed renders during weathering; this was especially evident in the deepest layers of the thermal insulation [110]. The test program used included the following approaches: heat–rain cycles for 20 days, heat–cold cycles for 5 days and rain–cold cycles for 20 days [110]. The high water absorption in the aerogel-based render caused its damage after undergoing the aforementioned freezing cycles, so an important issue in the future is to strengthen this layer of insulation and protect it from external moisture access. The applied ageing method did not affect the thermal conductivity coefficient, and no significant changes in it were recorded during the examined time. Similar relationships and conclusions were also presented by Berardi et al., who also studied the accelerated aging of lime-based aerogel composites under cyclic temperature changes, negative to −30 °C and positive to +40 °C, with different moisture content [106]. Other researchers also point out various moisture problems in the outer insulation layers, some of which seem to be important, such as the inability to dry completely over a long period of time or the phenomenon of condensation. A study by Ibrahim et al. revealed that the application of an additional insulation layer in the form of a silica aerogel-based render on an uninsulated building or on a building with existing interior insulation results in a reduction in or complete removal of the moisture problem [111]. Moreover, Maia et al. demonstrated that the application of an additional protective layer to aerogel reduces the negative effects of accelerated aging and results in increased durability of the mortars over a longer service life [113].

**Figure 8:**
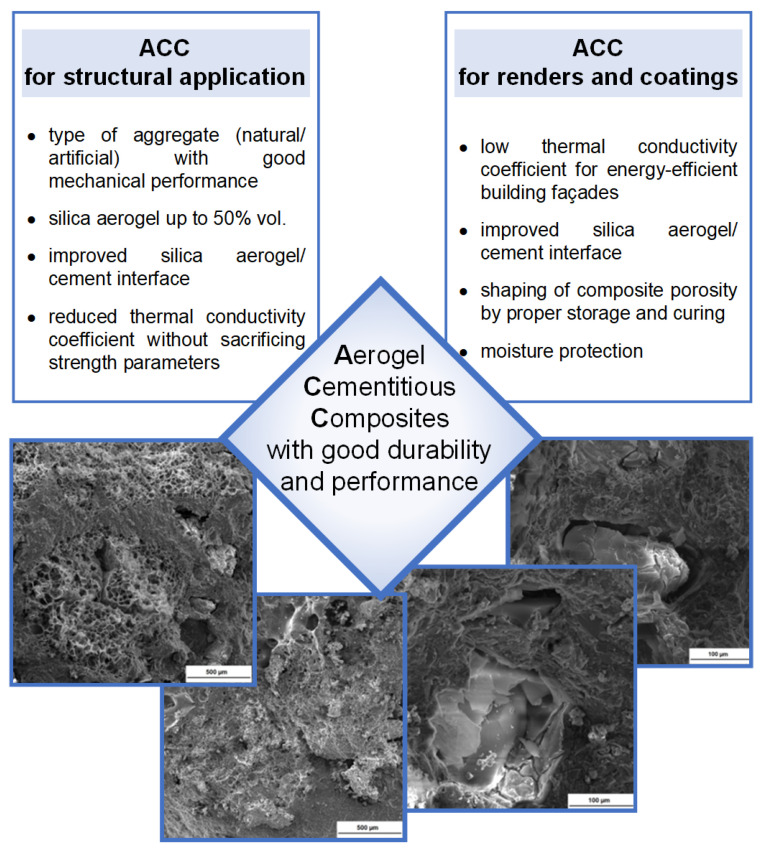
Development pathways for aerogel cement composites.

A summary of recent trends in aerogel cementitious composites depending on the potential use and the factors for durable high-performance materials that guarantee long service life is shown in Figure 8.

## 5. Conclusions

This paper collects and discusses recent publications on cementitious composites with silica aerogel. The unique properties of silica aerogel, such as its transparency, low density and high porosity, make this material an interesting solution for lowering the thermal conductivity of the cement matrix and creating new, eco-efficient future mortar and concrete materials that meet stringent energy conditions. The listed studies clearly indicate two directions in the development of aerogel cement composites. In the first, research is conducted with the aim of creating ultralight cement composites for lightweight façade systems, such as thermal renders with silica aerogel as an aggregate. These hold very good insulation parameters but sacrifice strength parameters. In the second, research is directed towards the creation of green, lightweight cement composites based on lightweight aggregates that come with good thermal conductivity and good strength parameters. Among these are cenospheres, fly ash-based aggregates and foam concrete. In these solutions, the aggregates are partially replaced by silica aerogel to further reduce the thermal conductivity while achieving satisfactory mechanical performance of the cement matrix.

This paper provides an analysis of the results of studies wherein cementitious composites were produced in the form of mortars and concretes in which silica aerogel was added as a replacement for natural or lightweight artificial aggregates. The density, strength and thermal conductivity relationships were assessed as a function of the amount of silica aerogel used. In addition, attention was paid to the aspects of water transport and porosity, which directly affect the durability of the composites studied. Based on the investigation, the following conclusions can be drawn: the introduction of silica aerogel into the cement matrix, usually at the expense of natural aggregate, contributes to a reduction in the density of the material by increasing the porosity, which in turn contributes to a significant reduction in the thermal conductivity coefficient. Nevertheless, the results show that large volume proportions of silica aerogel bring about significant decreases in compressive strength and increase the water absorbability of the cement composite. Therefore, an important aspect of future research on cement composites with silica aerogel will be to improve adhesion at the silica aerogel–cement matrix interface. Moreover, the issues of water transport and the durability of cement composites should be particularly studied in the coming years. These aspects will certainly contribute to the wider applicability of these materials in the construction industry.

## Figures and Tables

**Table 1:** The physical and mechanical properties of cementitious lightweight composites.

Aggregate Type/Maximum Size	Dry Density (kg/m^3^)	Compressive Strength (MPa)	Thermal Conductivity (W/(m·K))	References
Cenosphere/4 mm	1050–1350	5.0–30.1	0.46–0.60	[60]
Expanded perlite/2–4 mm	354–1833	0.1–28.8	0.06–0.13	[61]
Cenosphere/600 µm	1483–1890	44.3–48.1	0.29–0.37	[62]
Expanded glass/4 mm	1100–1380	23–30	0.49–0.85	[63]
Cenosphere/300 µm	1042–1300	40.9–69.4	0.31–0.40	[64]
Cenosphere/300 µm/GGBS (20–60%) in place of cement	1240–1270	Above 55	0.39–0.45	[65]
Cenosphere/500 µm	1282	52.5	0.6	[66]

**Table 2:** Classification of cementitious composites with silica aerogel in the literature.

Type of Cementitious Composite	Scientific Name of Cementitious Composites with Silica Aerogel	Silica Aerogel Volume (%)	References
Concretes	Ultra-high-performance aerogel concrete	20–80	[68]
	High-performance aerogel concrete	45–70	[69,70,71]
	Aerogel-incorporated concrete	10–60	[67,72]
	Ultra-lightweight concrete	15–60	[73]
Mortars	Silica aerogel-incorporated mortar	20–80	[74,75,76,77,78]
Lightweight Composites	Green lightweight composite	1–5	[62,79]
	Lightweight cement-based composite	15–100	[80,81]
	Ultra-lightweight cement composite	–	[73,82,83]
	Silica aerogel-incorporated composite	2–8	[84,85,86]
	Thermal renders with silica aerogel	0–20	[86,87]

**Table 3:** Thermal conductivity of cementitious composites with silica aerogel.

No.	Type of Cementitious Composite	Thermal Conductivity at 28 Days (W/(m·K))				References
		Silica Aerogel Content (vol%)				
		0	20	40	60	
1	Concrete	1.9	1.45	0.8	0.25	[67]
2	Concrete	2.3	1.5	0.8	0.5	[68]
3	High-performance concrete		0.35	0.20	0.19	[69]
4	High-performance concrete			0.28	0.20	[70]
5	High-performance concrete				0.26–0.14	[71]
6	Ultra-lightweight concrete		0.31–0.30			[73]
7	Structural concrete				1–0.86	[72]
8	Mortar			0.48	0.30	[74]
9	Mortar	1.2			0.2	[78]
10	Mortar	1.76	1.14	0.80	0.60	[77]
11	Lightweight composite		0.41–0.25			[79]
12	Lightweight composite		0.65–0.58			[85]
13	Lightweight composite				0.18	[81]
